# Prediction of vaginal birth after cesarean delivery in Chinese parturients

**DOI:** 10.1038/s41598-018-21488-6

**Published:** 2018-02-15

**Authors:** Juan Wen, Xuejing Song, Hongjuan Ding, Xiaofeng Shen, Rong Shen, Ling-qun Hu, Wei Long

**Affiliations:** 1 Nanjing Maternity and Child Health Care Institute, The Affiliated Obstetrics and Gynecology Hospital of Nanjing Medical University (Nanjing Maternity and Child Health Care Hospital), Nanjing, 210004 China; 2 State key Laboratory of Reproductive Medicine, The Affiliated Obstetrics and Gynecology Hospital of Nanjing Medical University (Nanjing Maternity and Child Health Care Hospital), Nanjing, 210004 China; 3 Department of Obstetrics, The Affiliated Obstetrics and Gynecology Hospital of Nanjing Medical University (Nanjing Maternity and Child Health Care Hospital), Nanjing, 210004 China; 4 Department of Anaesthesia, The Affiliated Obstetrics and Gynecology Hospital of Nanjing Medical University (Nanjing Maternity and Child Health Care Hospital), Nanjing, 210004 China; 5 Department of Anesthesiology, Northwestern University Feinberg School of Medicine, Chicago, IL 60611 USA

## Abstract

There is an urgent need in China to better predict vaginal birth after cesarean (VBAC) to face the challenge of the second child policy. We aimed to validate a widely used VBAC prediction model (Grobman’s model) and a modified version of this model in a Chinese population. In this retrospective cohort study, 444 women with one cesarean delivery and at least one subsequent attempt for a trial of labor in Nanjing, China were included. The considered potential VBAC predictors included Grobman’s background variables and five new variables. Overall, a total of 370 women had VBAC, with a success rate of 83.3%. The new background variables “maternal height” and “estimated fetal weight” were considered as two additional predictors for VBAC. The AUC of Grobman’s model was 0.831 (95%CI = 0.775–0.886) while the AUC of our modified model with two new variables added was 0.857 (sensitivity = 72.2%, specificity = 83.8%). However, the difference between the AUC of the two models was not significant (Z = −1.69, *P* = 0.091). We confirmed that Grobman’s model was accepted in the Chinese population. A modified model that is supplemented with maternal height and estimated fetal weight needs to be further studied in the Chinese population.

## Introduction

The cesarean delivery (CD) rate has increased substantially over recent decades. It is estimated that almost a third of women have delivered by CD worldwide^1,2^. Consequently, the number of pregnant women who had a prior CD has also increased each year. A recent study reported that the overall CD rate in China has been increasing from 28.8% in 2008 to 34.9% in 2014; particularly, the CD rates in 17 super cities were between 18.2% and 68.8%, with a median of 48.7% in 2008^3^. Pregnant women face the decision of having an elective repeat cesarean delivery (ERCD) or attempting a vaginal birth after cesarean delivery (VBAC), also called trial of labor after cesarean delivery (TOLAC)^4^. In October 2015, the universal two-child policy in China started, and healthcare providers have faced the biggest challenges of either a higher CD rate, a higher TOLAC rate, or both.

In an effort to decrease the CD rate with evidence of successful VBAC associated with less maternal and child morbidity and mortality, the National Institutes of Health (NIH), American College of Obstetricians and Gynecologists (ACOG), and Society for Maternal-Fetal Medicine (SMFM) recommended that TOLAC is a reasonable option for most pregnant women with a single prior CD^4,5^. However, unsuccessful TOLAC with intrapartum CD was associated with worse maternal and child clinical outcomes than those choosing ERCD^6–8^.

Predicting the chance of a successful TOLAC has been a clinically important topic since a successful TOLAC is associated with a decreased risk of future pregnancy complications and a shorter postpartum recovery time with fewer complications. Success rates of TOLAC were approximately between 60 and 90%^9,10^, and were associated with multiple factors. Any prior vaginal delivery, vaginal delivery after prior cesarean, cervical effacement, cervical dilation and station at admission are positively associated with TOLAC success. Conversely, advanced maternal age, recurring indication for cesarean, increased maternal body mass index (BMI), late estimated gestational age (EGA), preeclampsia and induction of labor are associated with TOLAC failure^11^. Maternal height and interval time from prior cesarean can also affect the success of TOLAC^12,13^. Thus, VBAC prediction models would be useful in clinical practice.

Several VBAC prediction models have been studied^11,14^. Some have been externally validated or supplemented with new variables^9,13,15^. Among those, Grobman’s model, which was developed based on the Maternal-Fetal Medicine Units (MFMU) Network, has been most commonly utilized and validated in a similar heterogeneous population of the United States, as well as among various geographic and ethnic cohorts including Japanese women; however, this model has not been utilized or validated in the Chinese population, the world’s biggest population^9,13,14,16–19^. Grobman’s model considered maternal age, pre-pregnancy BMI, race, prior vaginal delivery, prior VBAC, and indication for prior CD as factors that were obtainable at the first prenatal visit with supplemental variables appearing in late pregnancy or at admission for delivery^11^.

Chinese prenatal care has other unique features compared with a homogeneous population. Similar to racial disparity issues with medically underserved populations, population migrations have occurred from rural areas to cities, and from poor economic development regions to southeast regions, such as Nanjing, resulting from an economic imbalance after Chinese reform in the last several decades. This socioeconomic variety may be less when the population moves within a province, such as Jiangsu province where Nanjing is the capital city. A local prenatal registration system exists, and local citizens in Nanjing are more likely to have prenatal registrations with scheduled prenatal visits, indicating a higher socioeconomic status.

In this study, we aimed to validate the VBAC prediction model in a Chinese cohort and to determine potential modifications to optimize the model for this specific population.

## Results

**Figure 1:**
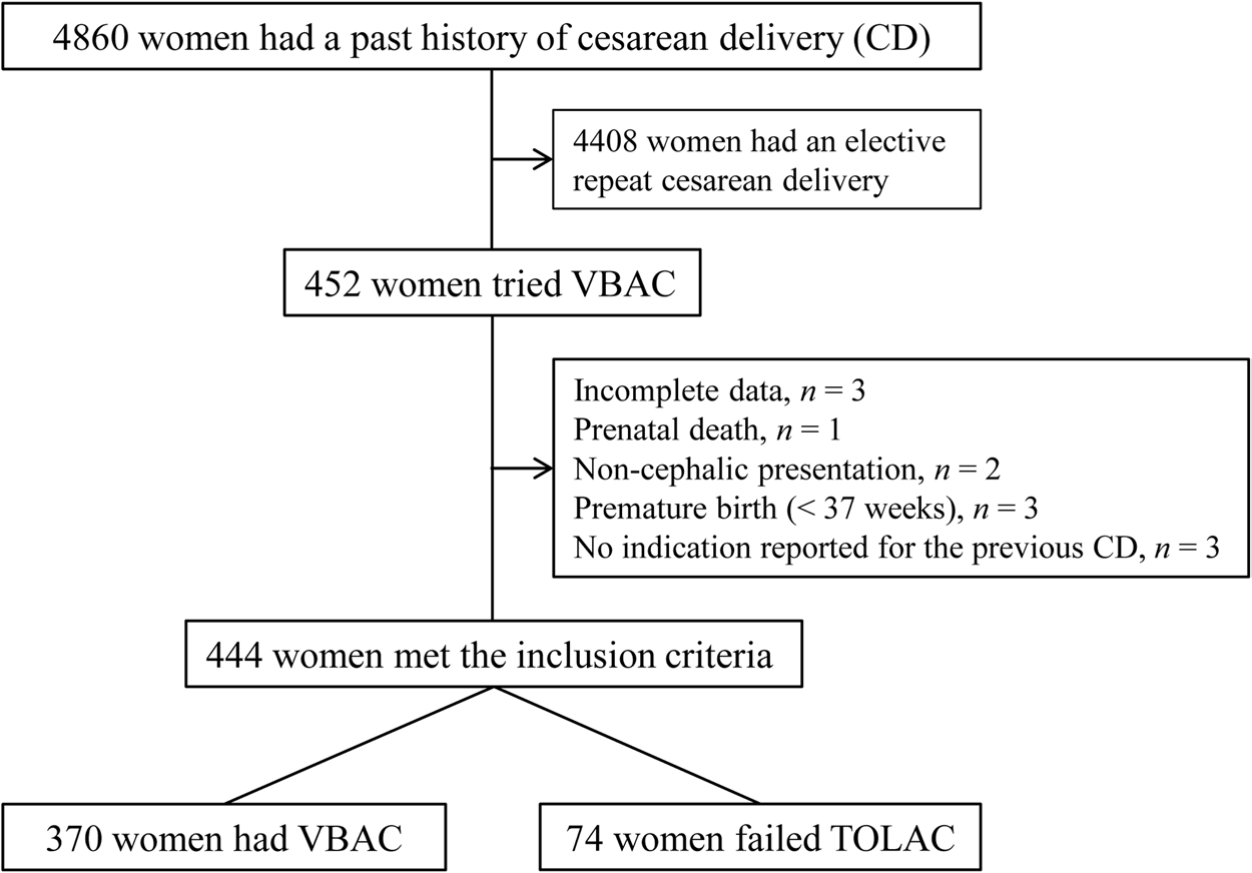
Flow-chart showing the study groups selection process.

During the study period, 4,860 women had a history of CD, and only 452 women tried VBAC; the rate of TOLAC was 9.3%. Four hundred and forty-four women (98.2%) met the inclusion criteria, and complete data were available for these women. The characteristics for these women are shown in Table 1. A total of 370 women had VBAC and 74 women failed TOLAC, with a success rate of 83.3% (Fig. 1). Compared with the failed TOLAC group, parturients in the successful group were less likely to have recurring indications for cesarean; additionally, these parturients were more likely to be taller, have a lower BMI at the last prenatal visit, have a lower estimated fetal weight, have a younger EGA at delivery, have lower rates of preeclampsia and labor induction, and have more cervical effacement and dilation at admission, and have a lower station at admission (all *P* < 0.05). The difference in the distribution of indications for the previous CD between the two groups was also significant (*P* < 0.05). However, there were no significant differences in the distribution of maternal age, maternal residence, any prior vaginal delivery, vaginal delivery after prior cesarean, interval time from prior cesarean, perinatal care registration, or labor analgesia rate between the successful and failed TOLAC groups (all *P* > 0.05).

**Table 1:** Characteristics of patients attempting a trial of labor.

Variable	Total (n = 444)	Success (n = 370)	Failed (n = 74)	*P* *
Maternal age (years)	31.40 ± 4.00	31.41 ± 4.10	31.34 ± 3.47	0.886
Maternal residence				0.834
Nanjing of Jiangsu province	341 (76.8)	285 (77.0)	56 (75.7)	
Other cities of Jiangsu province	22 (5.0)	19 (5.1)	3 (4.1)	
Other provinces	81 (18.2)	66 (17.8)	15 (20.3)	
Gravidity	3 (2, 4)	3 (2, 4)	3 (2, 4)	0.585
Parity	1 (1, 1)	1 (1, 1)	1 (1, 1)	0.963
Recurring indication for cesarean	9 (2.0)	4 (1.1)	5 (6.8)	0.007
Any prior vaginal delivery	37 (8.3)	32 (8.6)	5 (6.8)	0.591
Vaginal delivery after prior cesarean	15 (3.4)	14 (3.8)	1 (1.4)	0.481
BMI at last prenatal visit (kg/m^2^)	26.78 ± 2.87	26.49 ± 2.79	28.23 ± 2.83	<0.001
EGA at delivery (weeks)	38.78 ± 1.18	38.73 ± 1.19	39.03 ± 1.12	0.046
Preeclampsia	6 (1.4)	2 (0.5)	4 (5.4)	0.006
Cervical effacement at admission (10%)	9.28 ± 1.57	9.60 ± 1.06	7.68 ± 2.46	<0.001
Cervical dilation at admission (cm)	1.66 ± 1.49	1.84 ± 1.47	0.78 ± 1.27	<0.001
Station at admission (fifths scale)	3 (3, 3)	3 (3, 3)	3 (2, 3)	<0.001
Induction of labor	8 (1.8)	3 (0.8)	5 (6.8)	0.002
Maternal height	160.68 ± 4.25	160.94 ± 4.18	159.39 ± 4.39	0.006
Estimated fetal weight	3311.26 ± 379.82	3285.76 ± 375.30	3438.73 ± 379.13	0.002
Interval time from prior cesarean (months)	78.05 ± 38.30	77.53 ± 39.01	80.62 ± 34.64	0.527
Perinatal care registration	287 (64.6)	233 (63.0)	54 (73.0)	0.100
Labor analgesia	98 (22.1)	86 (23.2)	12 (16.2)	0.183
Indications for the previous CD				0.009
Social factors	127 (28.6)	105 (28.4)	22 (29.7)	
Malpresentation	98 (22.1)	81 (21.9)	17 (23.0)	
Macrosomia	23 (5.2)	15 (4.1)	8 (10.8)	
Abnormal labor stages	19 (4.3)	13 (3.5)	6 (8.1)	
Fetal distress	61 (13.7)	55 (14.9)	6 (8.1)	
Amniotic fluid volume abnormality	37 (8.3)	31 (8.4)	6 (8.1)	
Prolonged pregnancy, ≥42 weeks	20 (4.5)	16 (4.3)	4 (5.4)	
Severe pregnancy complications or maternal disease	29 (6.5)	24 (6.5)	5 (6.8)	
Cord around neck, ≥3 cycles	30 (6.8)	30 (8.1)	0 (0.0)	

BMI, body mass index; EGA, estimated gestational age; CD, cesarean delivery; Data are mean ± standard deviation, Median (IQR) or n (%). *χ^2^ test, Student-t test or Mann-Whitney test as appropriate.

The adjusted ORs for the original Grobman’s model variables that were obtained from the current study after multiple logistic regression analysis are shown in Table 2. The “any prior vaginal delivery” and “cervical effacement at admission” were both positively associated with VBAC (all *P* < 0.05). The “recurring indication for cesarean”, “BMI at last prenatal visit”, “preeclampsia” and “induction of labor” were all negatively associated with VBAC (all *P* < 0.05). Moreover, “vaginal delivery after prior cesarean” was positively associated with VBAC with borderline significance (*P* = 0.059). We added five new background variables (maternal height, estimated fetal weight, interval time from prior cesarean, perinatal care registration and labor analgesia) to the model (Table 2, right column). After stepwise regression analysis, “recurring indication for cesarean”, “any prior vaginal delivery”, “vaginal delivery after prior cesarean”, “BMI at last prenatal visit”, “preeclampsia”, “cervical effacement at admission”, “induction of labor”, “maternal height” and “estimated fetal weight” were entered into the VBAC prediction model. Both “maternal height” and “estimated fetal weight” were considered as two additional predictors for VBAC.

**Table 2:** Results of full model for vaginal birth after cesarean delivery after stepwise regression analysis, according to Grobman background variables [2009] supplemented with information on maternal height and estimated fetal weight. The multiple logistic regression models included all variables listed in the respective columns.

Variables	Grobman background variables estimated from the current study population		Grobman background variables supplemented with information on maternal height and estimated fetal weight	
	AOR (95%CI)	*P*	AOR (95%CI)	*P*
Variables selection from Grobman *et al*.^11^				
Maternal age (years)	0.935 (0.856–1.022)	0.139	0.942 (0.862–1.029)	0.186
Maternal residence				
Nanjing of Jiangsu province	1.000 (Ref)			
Other cities of Jiangsu province	1.549 (0.692–3.467)	0.287		
Other provinces	3.040 (0.543–17.006)	0.206		
Recurring indication for cesarean	0.097 (0.016–0.578)	0.010	0.081 (0.012–0.549)	0.010
Any prior vaginal delivery	3.993 (1.141–13.971)	0.030	4.468 (1.287–15.515)	0.018
Vaginal delivery after prior cesarean	12.934 (0.910–183.801)	0.059	19.513 (1.225–310.909)	0.035
BMI at last prenatal visit (kg/m^2^)	0.803 (0.720–0.894)	<0.001	0.824 (0.741–0.918)	<0.001
EGA at delivery (weeks)	1.016 (0.762–1.355)	0.913		
Preeclampsia	0.079 (0.011–0.574)	0.012	0.057 (0.007–0.472)	0.008
Cervical effacement at admission (10%)	1.771 (1.370–2.290)	<0.001	2.008 (1.665–2.422)	<0.001
Cervical dilation at admission (cm)	1.102 (0.752–1.614)	0.618		
Station at admission (fifths scale)	1.529 (0.677–3.454)	0.307		
Induction of labor	0.085 (0.008–0.934)	0.044	0.105 (0.010–1.098)	0.060
Additional background variables				
Maternal height			1.122 (1.040–1.211)	0.003
Estimated fetal weight			0.999 (0.998–1.000)	0.013
Performance, ROC AUC	0.831 (0.775–0.886)		0.857 (0.810–0.904)	

AOR, adjusted odds ratio; CI, confidence interval; BMI, body mass index; EGA, estimated gestational age; ROC AUC, area under the receiver operating characteristics curve.

**Figure 2:**
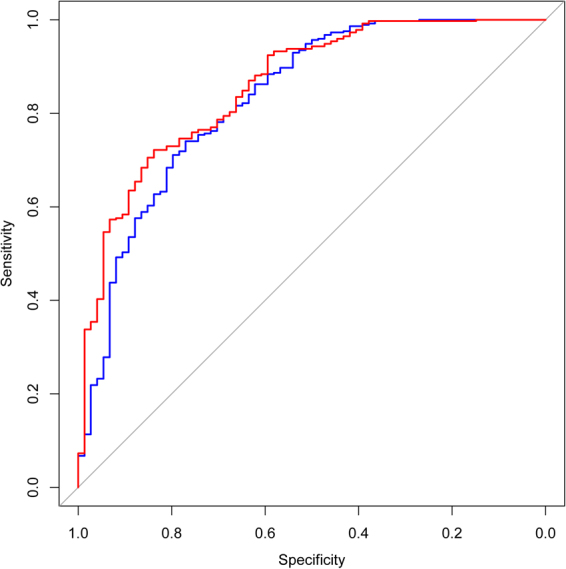
Receiver-operating characteristic (ROC) curves for the logistic regression model for prediction of VBAC success resulting from a trial of labor. Blue line, the panel based on Grobman background variables, including maternal age, maternal residence, recurring indication for cesarean, any prior vaginal delivery, vaginal delivery after prior cesarean, BMI at last prenatal visit, EGA at delivery, preeclampsia, cervical effacement at admission, cervical dilation at admission, station at admission and induction of labor (AUC = 0.831, sensitivity = 74.1%, specificity = 77.0%); Red line, the panel based on the results of full model for VBAC after stepwise regression analysis, including maternal age, recurring indication for cesarean, any prior vaginal delivery, vaginal delivery after prior cesarean, BMI at last prenatal visit, preeclampsia, cervical effacement at admission, induction of labor, maternal height and estimated fetal weight (AUC = 0.857, sensitivity = 72.2%, specificity = 83.8%). VBAC, vaginal birth after cesarean; BMI, body mass index; EGA, estimated gestational age; AUC, area under the curve.

**Figure 3:**
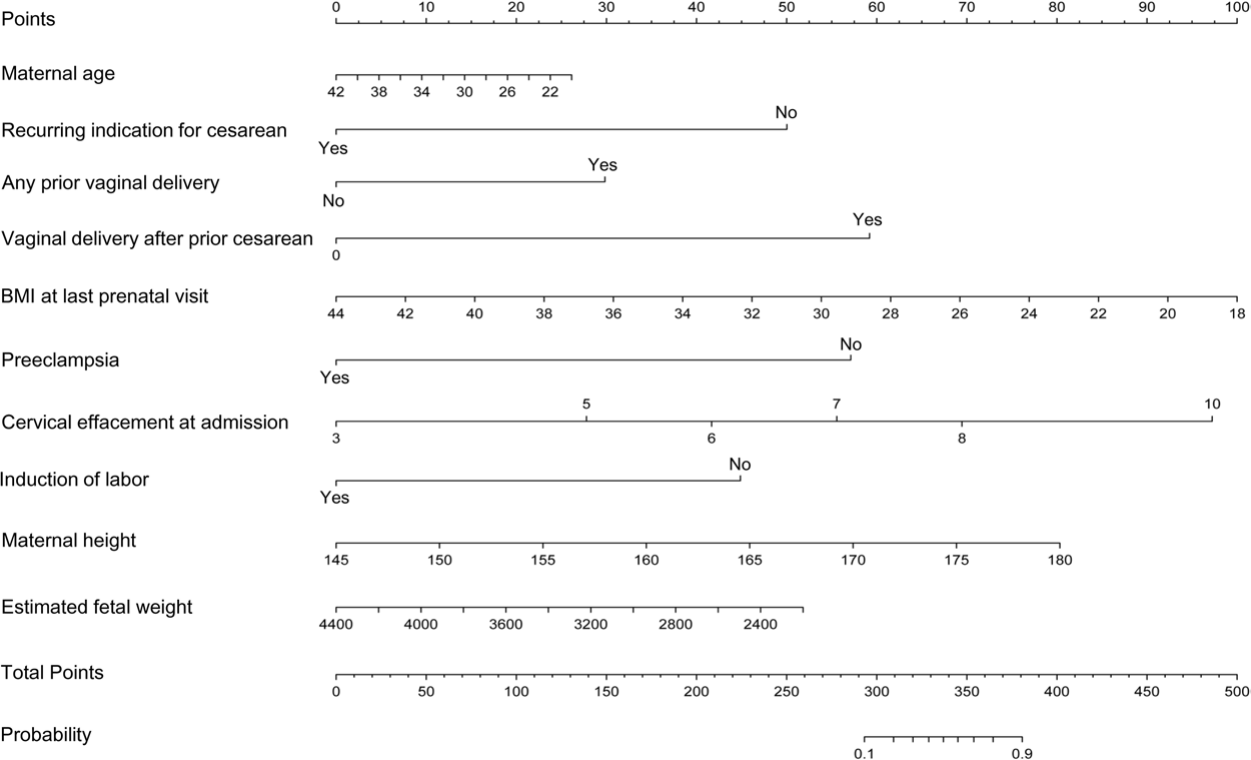
Predictive graphical nomogram for probability of vaginal birth after cesarean success resulting from a trial of labor.

Both the modified and original Grobman’s models were evaluated with constructed ROC curves for comparison with our data (Fig. 2). The AUC of Grobman’s model was 0.831 (95%CI = 0.775–0.886) while the AUC of our modified model with two new variables added was 0.857 (sensitivity = 72.2%, specificity = 83.8%). However, the difference between the AUC of the two models was not significant (Z = −1.69, *P* = 0.091). For the modified model, the Hosmer-Lemeshow χ^2^ (8 degrees of freedom) was 9.84 (*P* = 0.276), giving no cause for concern over model fit or calibration. The graphical nomogram for the modified model is presented in Fig. 3. Each patient characteristic is aligned with the corresponding number of points on the uppermost point scale. After all characteristics are considered, the user sums all points and aligns the sum on the “total points” line with the predicted probability of VBAC.

## Discussion

The current study evaluated the original Grobman’s model in the Chinese population for the first time with its modified model for a homogenous population with slightly better prediction of VBAC. Indeed, based on the comparisons using univariate analysis and between the original Grobman’s model and our modified model, we do see some differences as expected, but the modified model showed insignificant differences in our relatively small sample size.

It is not surprising to find differences between the studied American heterogeneous population and Asian relatively homogeneous parturients. Using the same Grobman’s model, the AUCs in the original American study, Japanese women, and current Chinese parturients are different, i.e., 0.81, 0.75 and 0.83, respectively^9^. Generalizing Grobman’s model in the Chinese population seems to be acceptable. In our investigation, the AUC of the modified model is improved to 0.86 from 0.81 with two additional parameters, maternal height and estimated fetal weight, without statistical significance. With further investigation, we found an insignificant influence of maternal age, maternal residence status, EGA at delivery, cervical dilation and station at admission that were used in Grobman’s model, while a significant influence of maternal height and estimated fetal weight were found in our studied population. These differences may be the result of institutions, population, race, the limited sample size of our study, or a combination of these factors. Thus, further studies incorporating diverse populations are warranted to validate and extend our findings.

The TOLAC rate has not been fully investigated in China because of limited numbers of patients as a result of the previous one-child policy. It was only 9.3% in our study; we expected more with the recent one-child policy that ended in October 2015. Patient safety is one of the biggest concerns overall; potential uterine rupture during TOLAC had a major negative impact in the US population. Team-based medicine and the availability of anesthesia services in Chinese labor and delivery suites have been addressed recently and will still take time to minimize this issue and face the challenges of an emerging need as a result of the second child policy with an existing high CD rate^20^. There are reasons for negative attitudes from some healthcare providers and counsellors concerning the rare but serious complications from TOLAC with fear of women’s refusal and litigation^21^. Without advanced practices, experiences of proactive labor epidural placement and anesthesia approaches, the consequences that occurred in the US population may recur. Women and their family members would not accept hysterectomy as a risk of her mode of birth. Therefore, better prediction of VBAC becomes one of the most important factors for promoting TOLAC.

It is well known that adverse maternal consequences, such as hysterectomy, bowel or bladder injury, transfusion, and placenta previa or accrete increased with multiple CD^22^. Additionally, the medical costs were also escalated with the increasing rate of CD. It is a particularly important public health issue in China with the combination of the currently high CD rate and newly issued universal two-child policy in 2015. TOLAC has several prominent advantages in the Chinese population with a vaginal childbirth option including quicker recovery and more importantly, avoidance of major abdominal surgery with less potential harmful disadvantages from unnecessarily repeated CD^4^. Therefore, TOLAC is widely recommended for the entire Chinese population in appropriately selected and supported pregnant women with one prior CD.

It might be regarded as an advantage that factors that were available at the first prenatal visit and factors appearing in late pregnancy or at admission for delivery were considered in our modified VBAC prediction model. Moreover, our population-based study was conducted for exploring use of an existing prediction tool, which will facilitate the application of this useful tool. However, this study had limitations as well. First, TOLAC leading to VBAC depends on good patient characteristics, care provider practices and patients’ attitudes about TOLAC. In this study, the data on care provider practices and patients’ attitudes were absent. Second, the relatively small sample size of the total population, especially for certain independent variables, may make the statistical power inadequate. Third, because the data of pregnancy complications was absent in this study, we could not calculate the cut-off value for TOLAC; this may limit its application. Further prospective multi-center studies with larger sample sizes involving care provider practices, patients’ attitudes about TOLAC and pregnancy complications may validate and improve the prediction accuracy.

In summary, Grobman’s model was accepted in the Chinese population, and the model supplemented with information on maternal height and estimated fetal weight needs to be further investigated for the Chinese population before it’s application. The model provides an individual chance of VBAC for pregnant women considering TOLAC, and helps them make a more rational decision regarding delivery mode. Finally, it should be emphasized that both obstetricians and pregnant women should weigh the maternal and neonatal risks with benefits associated with TOLAC in a team-based medical setting.

## Materials and Methods

This is a retrospective cohort study. The study was approved by the institutional review board of Nanjing Maternity and Child Health Care Hospital, and the methods were carried out in accordance with the approved guidelines.

We identified women in the Nanjing Maternity and Child Health Care Hospital, where the annual delivery rate is approximately 20,000. These women would have given birth at least twice between 2006 and 2016, including one CD and at least one subsequent TOLAC. The last delivery was considered as the index delivery. For the pregnant women who had a prior CD, the intention to undergo a TOLAC or CD is enquired at the prenatal visit at 36 weeks; whether to perform a TOLAC depends on a comprehensive evaluation before the delivery. Written informed consent was obtained from all participants at the first prenatal visit. Data from electronic standardized medical records were used. The inclusion criteria and study variables were selected to validate the Grobman prediction model plus others for potential optimization of the model for Chinese women. These variables include: maternal age (years), maternal residence status (Nanjing of Jiangsu province, other cities of Jiangsu province or other provinces, corresponding to “maternal race” in the Grobman’s model), recurring indication for cesarean (yes/no), any prior vaginal delivery (yes/no), vaginal delivery after prior cesarean (yes/no), BMI at last prenatal visit (kg/m^2^), EGA at delivery (weeks), preeclampsia (yes/no), cervical effacement at admission (10%), cervical dilation at admission (cm), station at admission (fifths scale) and induction of labor (yes/no), similar to Grobman’s study^11^. We also considered maternal height (cm), estimated fetal weight (g), interval time from prior cesarean (months), perinatal care registration (yes/no) and labor analgesia (yes/no) in our potential model. The exclusion criteria were also the same as those in Grobman’s study, including prenatal death, non-cephalic presentation, premature birth (<37 weeks), multiple pregnancies, ERCD and no indication reported for the previous CD^14^.

The differences of the maternal and infant characteristics between the successful and failed TOLAC groups were calculated by Student’s *t*-test (for continuous variables) and *χ*^2^ test (for categorical variables). All estimates were calculated using multiple logistic regression analysis by computing odds ratios (ORs) and their 95% confidence intervals (CIs). The applicability of the variables for Grobman’s model was analysed in our Chinese data set. Similar to Grobman’s model, our modified prediction model for a successful TOLAC was constructed according to the following steps^23^: (1) Prediction factor selection: Grobman background variables (maternal age, maternal residence, recurring indication for cesarean, any prior vaginal delivery, vaginal delivery after prior cesarean, BMI at last prenatal visit, EGA at delivery, preeclampsia, cervical effacement at admission, cervical dilation at admission, station at admission and induction of labor) supplemented with information on maternal height, estimated fetal weight, interval time from prior cesarean, perinatal care registration and labor analgesia. (2) Model construction: the variables that remained in the forward stepwise model (with a significance level of 0.10 for entering and 0.11 for removing the respective explanatory variables) were included, and the prediction model was constructed using a logistic regression model. (3) Model evaluation: the model performance was evaluated by receiver-operator characteristic (ROC) curves, and the area under the curve (AUC) was used to classify the successful and failed TOLAC groups. The sensitivity and specificity were calculated to illustrate the model effects using the “best threshold” criteria of the ROC curve. The difference in the area under two correlated ROC curves was evaluated by DeLong’s test. The model’s calibration was assessed by Hosmer-Lemeshow χ^2^ test. A graphical nomogram was also produced for the new model so that the individual-specific probabilities of VBAC could be easily approximated. All the statistical analyses were performed with R software (version 3.3.0), and *P* ≤ 0.05 in a two-sided test was considered statistically significant.
